# Seropositivity of Transfusion-Transmissible Infections Among Blood Donors in the Eastern Zone of Tanzania During the Early COVID-19 Period: A Cross-Sectional Analysis

**DOI:** 10.7759/cureus.100450

**Published:** 2025-12-30

**Authors:** Magdalena Lyimo, Hafidhi H Ntissi, Abdu B Juma, Raphael Z Sangeda

**Affiliations:** 1 Department of Haematology and Blood Transfusion, Muhimbili University of Health and Allied Sciences, Dar es Salaam, TZA; 2 Department of Pharmaceutical Microbiology, Muhimbili University of Health and Allied Sciences, Dar es Salaam, TZA; 3 National Blood Transfusion Service, Ministry of Health, Dar es Salaam, TZA

**Keywords:** blood safety, covid-19, hepatitis b virus, hepatitis c virus, hiv, syphilis, tanzania, transfusion-transmissible infections

## Abstract

Background

Transfusion-transmissible infections (TTIs), such as human immunodeficiency virus (HIV), hepatitis B virus (HBV), hepatitis C virus (HCV) and syphilis, remain critical threats to blood safety in many low and middle-income countries. The COVID-19 pandemic has disrupted blood donation activities globally, raising concerns about its impact on donor screening outcomes. This study assessed TTI seropositivity among blood donors in the Eastern Zone of Tanzania during the early COVID-19 period.

Methods

A retrospective cross-sectional analysis was conducted using donor records collected between January and July 2020 at the Eastern Zone of the Blood Transfusion Centre. Screening adhered to the national and WHO-recommended algorithms using chemiluminescent immunoassays. Donor characteristics and TTI prevalence were summarized descriptively. Logistic regression was used to identify factors associated with HBV seropositivity, as hepatitis B had the highest prevalence and sufficient event counts to support stable multivariable analysis compared with other TTIs.

Results

Among 16,767 donors with complete records, 1,774 (10.7%) tested positive for at least one TTI. HBV was the most frequent infection (897, 5.4%), followed by syphilis (453, 2.7%), HIV (311, 1.9%), and HCV (207, 1.2%). Coinfections were rare: HBV-HIV (35, 0.21%), HBV-syphilis (27, 0.16%), and HBV-HCV (15, 0.09%). Blood collection volumes decreased sharply in April-May 2020 but recovered by July. Monthly HBV and TTI seroprevalence showed stable descriptive trends (4.1-6.1%), with no apparent increase during low-donation months. Independent predictors of HBV seropositivity included male sex (AOR 1.7, 95% CI 1.4-2.3) and age 30-39 years (AOR 3.2, 95% CI 2.1-4.8).

Conclusion

Despite disruptions in blood collection, TTI seroprevalence remained stable during the early COVID-19 period, suggesting resilience in donor selection and screening procedures. Strengthening donor recruitment and maintaining rigorous laboratory screening remain essential to safeguard the blood supply in Tanzania.

## Introduction

A safe and adequate blood supply is essential for effective health systems [1-3]; however, transfusion-transmissible infections (TTIs), including human immunodeficiency virus (HIV), hepatitis B virus (HBV), hepatitis C virus (HCV), and syphilis, continue to pose significant risks, particularly in many low and middle-income countries. The World Health Organization (WHO) recommends universal blood screening as a cornerstone of transfusion safety [2]. While improvements in blood safety have been achieved across Africa, persistent challenges, such as high background infection rates, inconsistent donor recruitment, and limited laboratory capacity, continue to affect national blood services [4,5].

Tanzania has strengthened its National Blood Transfusion Service (NBTS) through expanded blood collection, quality-assured screening, and improved regional coordination. Nonetheless, donor TTIs seroprevalence remains a public health concern, particularly in high-burden areas such as Dar es Salaam and the surrounding Eastern Zone [6,7]. The Eastern Zone, comprising Dar es Salaam, Pwani, Morogoro, and Dodoma, is the country’s largest blood-collection catchment area and therefore a key region for monitoring transfusion safety [6,7].

The COVID-19 pandemic, which began in early 2020, disrupted blood donation systems worldwide, with many countries reporting reduced donor turnout, interrupted mobilization activities, and difficulties sustaining an adequate blood supply [8,9]. Similar disruptions have been documented in parts of the African region, although the magnitude varies across countries [10]. Maintaining essential transfusion services during such periods requires resilient donor recruitment and robust screening [2,11].

Despite these global reports, there is limited data on TTI patterns in sub-Saharan Africa during the early phase of the COVID-19 pandemic [6,7]. Addressing this gap is important for understanding the stability of donor infection profiles and assessing the resilience of national blood systems during public health emergencies.

This study assessed the seropositivity of HIV, HBV, HCV, and syphilis among blood donors in the Eastern Zone of Tanzania between January and July 2020, coinciding with the implementation of the first national COVID-19 control measures. Secondary analyses examined co-infection patterns and temporal variations during this early pandemic period. The findings provide insights into the resilience of blood safety systems during health service disruptions and inform preparedness for future emergencies.

## Materials and methods

Study design and setting

This retrospective cross-sectional study analyzed routine blood donor data collected between January and July 2020 at the Eastern Zone Blood Transfusion Centre (EZBTC) in Dar es Salaam, Tanzania. The EZBTC coordinates donor mobilization, blood collection, and screening for TTIs across four regions of the Eastern Zone: Dar es Salaam, Pwani, Morogoro, and Dodoma.

Study population

The study population included all whole-blood donations collected during the seven-month study period. Donor eligibility was determined according to the National Blood Transfusion Service (NBTS) of Tanzania criteria, including age 18-65 years, body weight ≥50 kg, acceptable haemoglobin levels, and the absence of known behavioral or clinical risk factors for TTIs.

Data processing

A total of 42,017 donation records were extracted from the EZBTC database for analysis. Records were reviewed for completeness, and 16,767 donations with complete demographic and TTI screening data were retained. Excluded entries lacked key donor characteristics or laboratory results and were removed in accordance with standard NBTS data quality procedures. Descriptive review of the retained and cleaned datasets showed comparable patterns of TTI seropositivity.

Serological testing for TTIs

Serological screening followed the national TTI testing algorithm in accordance with WHO recommendations. HIV-1/2 antibodies, Hepatitis B surface antigen (HBsAg) and hepatitis C virus (HCV) antibodies were screened using chemiluminescent immunoassays. Syphilis screening was also performed by chemiluminescent assays, and a final confirmatory test was performed using rapid plasma reagin (RPR) and/or treponemal-specific assays. All initially reactive samples underwent duplicate repeat testing and confirmatory testing, and the final TTI status was determined based on the confirmatory results.

Statistical analysis

Descriptive statistics were used to summarize donor demographic characteristics and TTI seroprevalence. Associations between categorical donor characteristics and HBV serostatus were assessed using Pearson’s chi-square test, with the chi-square statistic (χ²), degrees of freedom (df), and p-values reported. Variables that demonstrated statistically significant associations (p< 0.05) in the chi-square analysis were subsequently included in a multivariable logistic regression model to identify factors independently associated with HBV seropositivity. Hepatitis B was selected for multivariable analysis because it had the highest prevalence and sufficient event counts to support stable adjusted estimates. Adjusted odds ratios (AORs) with corresponding 95% confidence intervals (CIs) were calculated. All analyses were conducted using SPSS (IBM SPSS Statistics for Windows, Version 26.0. Armonk, NY: IBM Corp.; 2019), and only complete records were included in the final analysis.

Ethical clearance

Ethical approval for this study was obtained from the Muhimbili University of Health and Allied Sciences (MUHAS) Institutional Review Board (Ref: DA.25/111/05/10/Feb/2020). Permission to access and analyze donor records was granted by the National Blood Transfusion Service (NBTS). The study used de-identified, archived donor data; therefore, informed consent was not required, as no personal identifiers were collected and the data were obtained initially under prior consent procedures.

## Results

Donor characteristics

**Figure 1:**
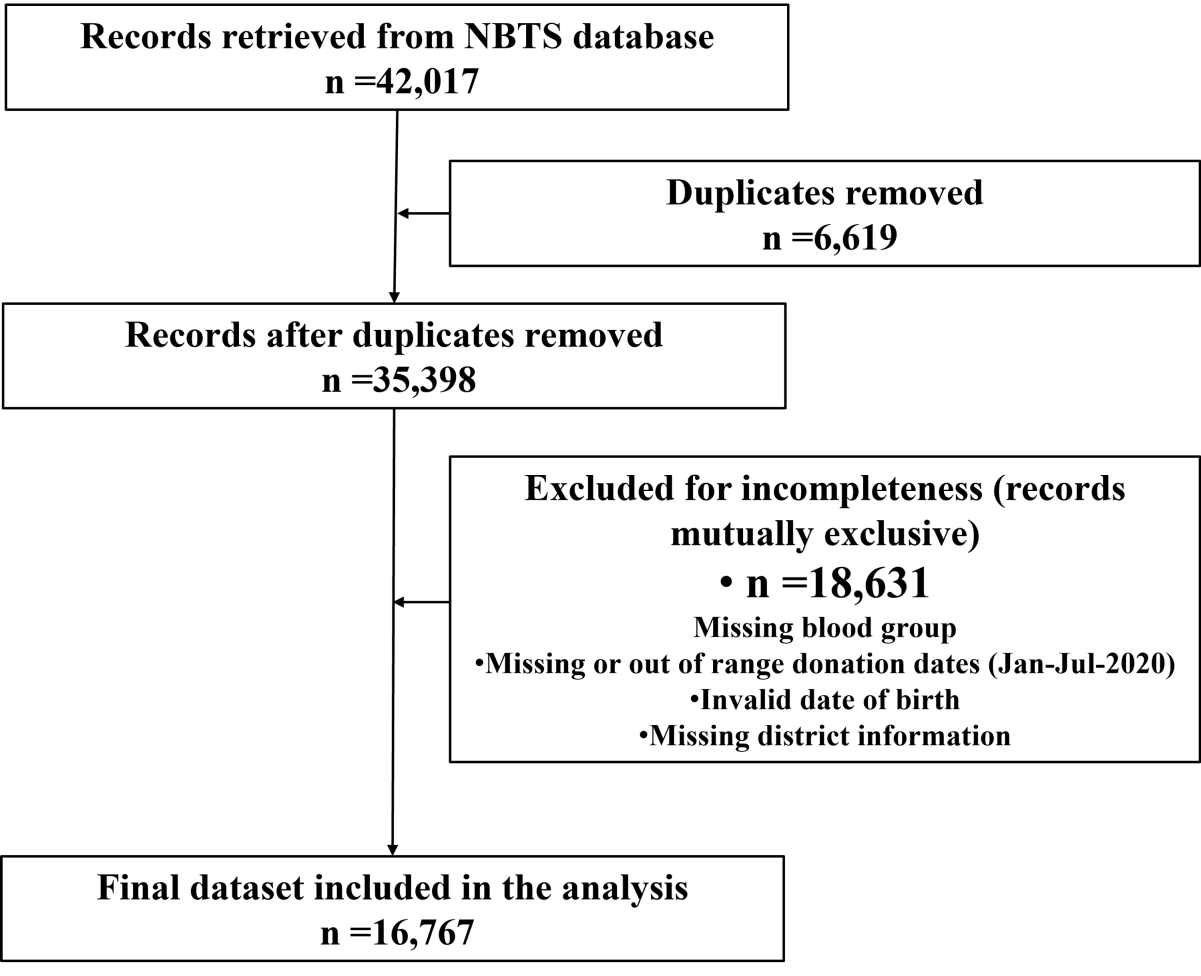
Data extraction and cleaning flowchart. Sequential, mutually exclusive filters were applied to the initial 42,017 donor records, removing duplicates and entries with missing blood group, missing or out-of-range donation dates, incomplete marital status, invalid date of birth, or missing district information. After stepwise exclusions, 16,767 complete donor records remained for analysis. Key: NBTS: National Blood Transfusion Service.

A total of 16,767 complete whole-blood donor records were analyzed after cleaning the initial 42,017 collected units (Figure 1).

Most donors were male (14,317, 85.4%), and the largest age group was 20-29 years (6,508, 38.8%). Secondary school students contributed 6,370 donations (38.0%), followed by farmers (4,302, 25.7%) and businessmen (3,776, 22.5%). Half of all units originated from Dar es Salaam (8,460, 50.5%). Blood group O-positive was the most frequent (8,791, 52.4%) (Table 1).

**Table 1:** Sociodemographic information and blood group distribution of whole blood units collected from the Eastern Zone (January-July 2020, n=16,767). EZBTC = Eastern Zone Blood Transfusion Center

Characteristics	Categories	Number	Percentage (%)
Gender	Male	14,317	85.4
	Female	2,450	14.6
Age (years)	<20	1,858	11.1
	20-29	6,508	38.8
	30-39	4,808	28.7
	40-49	2,726	16.3
	>50	867	5.2
Marital status	Divorced/separated	54	0.3
	Married	9,067	54.1
	Single	7,646	45.6
Occupation	Farmer	4,302	25.7
	Teacher	405	2.4
	College student	1,530	9.1
	Businessman	3,776	22.5
	Police	152	0.9
	Secondary student	6,370	38
	Soldier	232	1.4
Region	Dar es Salaam	8,460	50.5
	Dodoma	2,173	13
	Morogoro	3,622	21.5
	Pwani	2,515	15
Rhesus Positive	A	3,974	23.7
	AB	615	3.7
	B	3,160	18.8
	O	8,791	52.4
Rhesus Negative	A	57	0.3
	AB	8	0
	B	42	0.3
	O	120	0.7
Samples from other collection sites	-	11,656	69.5
Donor category (EZBTC, n=5,111)			
	Voluntary	3,378	66.1
	Replacement	1,733	33.9

Overall TTI seroprevalence

**Figure 2:**
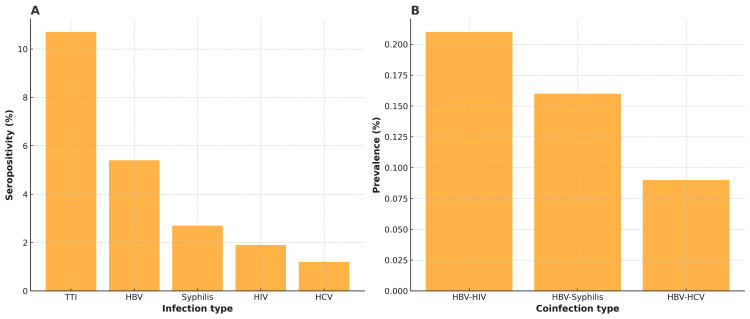
Overall seropositivity and hepatitis B virus (HBV) coinfection patterns among blood donors in the Eastern Zone of Tanzania, January-July 2020. Panel A: Seroprevalence of human immunodeficiency virus (HIV), HBV, hepatitis C virus (HCV), syphilis, and overall transfusion-transmissible infections (TTIs) in a clean dataset (n=16,767). Panel B: Prevalence of HBV coinfections: HBV-HIV, HBV-syphilis, and HBV-HCV expressed as the proportion of all donors with complete data (n=16,767).

Among all donors, 1,774 of 16,767 (10.7%) tested positive for at least one transfusion-transmissible infection (TTI). Hepatitis B was the most prevalent infection (897, 5.4%), followed by syphilis (453, 2.7%), HIV (311, 1.9%), and hepatitis C (207, 1.2%). Because some donors had more than one infection, the summed counts of individual TTIs exceed the number of unique seropositive donors (Appendix 1, Figure 2A). Monthly HBV and overall TTI prevalence remained descriptively stable from January to July, with only minor month-to-month variation in both the raw and cleaned datasets.

HBV-related coinfections were rare (Figure 2B). HBV-HIV coinfection constituted 0.21% of all donors (35 cases), followed by HBV-syphilis at 0.16% (27 cases) and HBV-HCV at 0.09% (15 cases). These findings indicate that coinfections contributed only a small proportion to the overall TTI burden.

**Figure 3:**
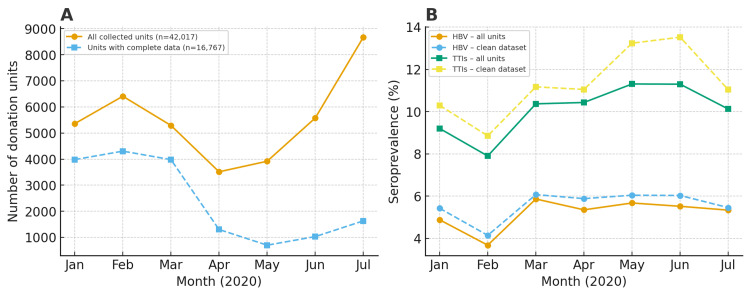
Trends of monthly blood donation volumes and seroprevalence of transfusion-transmissible infections (TTIs) in the Eastern Zone of Tanzania, January to July 2020. A: Monthly whole-blood collection volumes comparing all collected units (raw dataset, n=42,017) with units with complete screening and demographic information (clean dataset, n=16,767); B: Monthly seroprevalence of hepatitis B virus (HBV) and overall TTIs (combined HIV, HBV, Hepatitis C virus (HCV) and syphilis) plotted separately for the raw dataset (n=42,017) and the clean dataset (n=16,767), over seven months.

These trends are illustrated in Figures 3A, 3B, with detailed numerical values provided in Appendices 2, 3.

HBV seropositivity

A total of 897 donors (5.4%) were HBV-positive. Prevalence was higher among males (5.8%) than among females (2.8%, p< 0.001). Donors aged 30-39 years had the highest prevalence (345/4,808, 7.2%), followed by those aged 40-49 years (171/2,726, 6.3%). Married donors had a higher positivity rate (613/9,067, 6.8%) than single donors (282/7,646, 3.7%; p< 0.001). Farmers recorded the highest occupational prevalence (303/4,302, 7.1%), whereas soldiers had the lowest (6/232, 2.6%). Regionally, Pwani had the highest HBV seroprevalence (161/2,515, 6.5%) and Dodoma the lowest (104/2,173, 4.8%) (Table 2).

**Table 2:** Sociodemographic factors associated with hepatitis B virus (HBV) seropositivity.

Characteristic	Category	HBV-positive n (%)	χ² (df), p-value
Gender	Male	828 (5.8)	36.84 (1), p< 0.001
	Female	69 (2.8)	
Age (years)	<20	29 (1.6)	94.49 (4), p< 0.001
	20-29	305 (4.7)	
	30-39	345 (7.2)	
	40-49	171 (6.3)	
	≥50	47 (5.5)	
Marital status	Single	282 (3.7)	77.68 (2), p< 0.001
	Married	613 (6.8)	
	Divorced/separated	2 (3.8)	
Occupation	Secondary student	282 (4.5)	56.65 (6), p< 0.001
	Farmer	303 (7.1)	
	Businessman	223 (5.9)	
	College student	50 (3.3)	
	Teacher	21 (5.2)	
	Police	12 (7.9)	
	Soldier	6 (2.6)	
Region	Dar es Salaam	422 (5.0)	10.93 (3), p=0.012
	Dodoma	104 (4.8)	
	Morogoro	210 (5.9)	
	Pwani	161 (6.5)	

HBV coinfections

HBV-related coinfections were uncommon. HBV-HIV coinfection occurred in 35 donors (0.21%), HBV-syphilis in 27 (0.16%), and HBV-HCV in 15 (0.09%). These patterns are presented in Appendix 4.

Multivariate predictors of HBV seropositivity

In multivariate logistic regression (Table 3), male donors had higher odds of HBV infection (AOR 1.7; 95% CI 1.4-2.3). Age was strongly associated with HBV positivity, with the highest adjusted risk seen in donors aged 30-39 years (AOR 3.2; 95% CI 2.1-4.8), followed by ages 20-29 (AOR 2.4; 95% CI 1.63-3.6) and 40-49 (AOR 2.6; 95% CI 1.7-4.1). Married donors also had significantly higher odds of HBV seropositivity (AOR 1.3; 95% CI 1.1-1.6). After adjustment, occupation and region were not significant predictors.

**Table 3:** Factors associated with hepatitis B virus seropositivity (logistic regression). cOR: crude odds ratio; CI: confidence interval; AOR: adjusted odds ratio.

Characteristic	Category	cOR (95% CI)	p-value	AOR (95% CI)	p-value
Gender	Female	Ref	-	Ref	-
	Male	2.1 (1.7-2.7)	<0.0001	1.7 (1.4–2.3)	<0.0001
Occupation	Secondary student	Ref	-	Ref	-
	Farmer	1.6 (1.4-1.9)	<0.0001	1.2 (0.99-1.5)	0.057
	Businessman	1.4 (1.1-1.6)	0.001	1.0 (0.85-1.2)	0.796
	College student	0.73 (0.54-0.99)	0.044	0.78 (0.57-1.1)	0.135
	Teacher	1.18 (0.75-1.9)	0.475	0.93 (0.59-1.5)	0.771
	Soldier	0.58 (0.26-1.3)	0.193	0.51 (0.23-1.2)	0.119
	Police	1.8 (1.0-3.4)	0.044	1.4 (0.77-2.6)	0.261
Marital status	Single	Ref	-	Ref	-
	Married	1.9 (1.6-2.2)	<0.0001	1.3 (1.1-1.6)	0.001
	Divorced/separated	1.0 (0.24-04.2)	0.982	0.77 (0.2-3.2)	0.723
Age group	<20	Ref	-	Ref	-
	20-29	3.1 (2.1-4.6)	<0.0001	2.4 (1.63-3.6)	<0.0001
	30-39	4.9 (3.3-7.1)	<0.0001	3.2 (2.1-4.8)	<0.0001
	40-49	4.2 (2.8-6.3)	<0.0001	2.6 (1.7-4.1)	<0.0001
	>50	3.6 (2.3-5.8)	<0.0001	2.3 (1.4-3.8)	0.002
Region	Pwani	Ref	-	Ref	-
	Dar es Salaam	0.76 (0.63-0.91)	0.004	0.93 (0.75-1.2)	0.571
	Dodoma	0.74 (0.57-0.95)	0.018	0.75 (0.58-0.98)	0.033
	Morogoro	0.90 (0.73-1.1)	0.33	0.88 (0.71-1.1)	0.274
Donation category	Voluntary	Ref	-	Ref	-
	Replacement	1.34 (1.02-1.77)	0.037	1.05 (0.78-1.40)	0.755

Blood collection trends

Monthly blood collection declined sharply during April and May 2020. In the raw dataset, volumes fell from 5,359 units in January to 3,514 in April and 3,918 in May; the clean dataset showed similar decreases from 3,981 in January to 1,301 in April and 700 in May. Collection volumes recovered in June and increased further in July (Figure 3A and Appendix 5).

## Discussion

This study provides substantial evidence of transfusion-transmitted infections in the Eastern Zone of Tanzania during the first months of the COVID-19 pandemic. Although blood collection volumes decreased sharply in April and May 2020, the overall TTI seroprevalence remained stable over the following 7 months. This suggests that donor selection, laboratory screening, and quality assurance procedures remained functional despite service disruptions, an observation consistent with reports from other settings where structured screening algorithms helped maintain blood safety during the pandemic [2,8].

HBV and syphilis were the most frequently detected TTIs, consistent with evidence from Tanzania and other sub-Saharan African countries [7,12,13]. The predominance of male donors reflects the longstanding sociocultural and logistical patterns in the region [14]. The higher HBV prevalence among donors aged 30-39 years and those who were married is consistent with established epidemiological risk profiles in East Africa and globally [13,15]. Although regional differences were observed at the descriptive level, they did not persist after adjustment, suggesting that demographic factors, rather than geography, were stronger drivers of infection risk.

Notably, the stability of monthly TTI patterns suggests that the reduction in donor numbers was not associated with an increase in TTI seroprevalence. Similar findings have been reported in other countries, where donation volumes decreased, but seropositivity remained unchanged, underscoring the resilience of the national screening systems [9,10,16]. These results are consistent with the importance of robust donor selection criteria, reliable confirmatory testing, and strong coordination between regional transfusion centres. Strengthening these systems is essential to ensure blood safety during future public health emergencies.

Limitations

This study has some limitations. First, as a retrospective analysis of routine blood donation surveillance data, the findings depended on the accuracy and completeness of donor records. A large proportion of records were excluded due to missing key demographic or laboratory variables, which may have introduced selection bias that cannot be fully excluded in this analysis. Second, blood donors represent a generally healthier and lower-risk population than the general public; therefore, the observed prevalence of transfusion-transmissible infections (TTIs) likely underestimates population-level prevalence. Third, behavioral risk factors were not available in the dataset, limiting the ability to assess individual-level risk determinants beyond sociodemographic characteristics. Fourth, the analysis was restricted to the Eastern Zone of Tanzania, and the findings may not be fully generalizable to other regions with different donor demographics or epidemiological profiles. Finally, the study covered only the first seven months of 2020; long-term trends beyond the early COVID-19 period were not assessed.

## Conclusions

TTI seroprevalence in the Eastern Zone of Tanzania remained substantial during the early COVID-19 period, with HBV contributing to the highest burden. Despite pronounced reductions in blood collection during April and May 2020, the screening process remained intact, and no increase in donor infection risk was observed. These findings underscore the importance of maintaining strong donor recruitment, rigorous screening algorithms, and continuous surveillance. Strengthening risk-based donor selection and expanding public health education will further support safe and sustainable blood collection in Tanzania.

## Appendices

Appendix 1

To support the monthly trends shown in Figures 2, 3, additional numerical summaries are provided in the following tables (Appendix 1-5; Tables 4-8). Table 4 summarizes overall seropositivity for individual infections (HIV, Hepatitis B Virus (HBV), Hepatitis C Virus (HCV) and syphilis) and combined transfusion-transmissible infections (TTIs). Table 5 and Table 6 present the monthly HBV and overall TTI seroprevalence, respectively. Table 7 shows the distribution of HBV-related coinfections and Table 8 lists monthly whole-blood donation volumes for both the raw and clean datasets.

**Table 4:** Seropositivity for individual infections (HIV, HBV, HCV, syphilis) and combined TTIs among donors with complete data (n=16,767). HBV: Hepatitis B Virus; HCV: Hepatitis C Virus.

Infection	Positive N	Positive %	Negative N	Total N
HBV	897	5.4	15,754	16,651
Syphilis	453	2.7	16,172	16,625
HIV	311	1.9	16,327	16,638
HCV	207	1.2	16,442	16,649
Any TTI	1,774	10.7	14,817	16,591

Appendix 2 

**Table 5:** Monthly HBV seroprevalence in the raw and clean datasets, January-July 2020.

Month	HBV positive (clean)	Total clean	HBV % (clean)	HBV % (raw)
January	216	3,981	5.4	5.1
February	177	4,271	4.1	4.2
March	237	3,903	6.1	5.8
April	74	1,258	5.9	6.0
May	42	695	6.0	5.9
June	62	1,028	6.0	6.1
July	89	1,631	5.5	5.6

Appendix 3* *

**Table 6:** Monthly overall transfusion-transmissible infection (TTI) seroprevalence in the raw and clean datasets, January-July 2020.

Month	TTI % (raw)	TTI % (clean)*
January	10.4	10.7
February	10.2	10.3
March	11	11.4
April	10.7	10.9
May	11.2	11.5
June	10.8	11.2
July	10.4	10.7

Appendix 4 

**Table 7:** Prevalence of HBV-related coinfections (HBV-HIV, HBV-syphilis, HBV-HCV) among donors with complete data (n=16,767).

Coinfection pair	Positive n	Proportion of total donors (%)
HBV–HIV	35	0.21
HBV–Syphilis	27	0.16
HBV–HCV	15	0.09

Appendix 5 

**Table 8:** Monthly whole-blood donation volumes in the raw (n=42,017) and clean (n=16,767) datasets, January-July 2020.

Month	Raw donations (n)	Clean donations (n)
January	5,359	3,981
February	6,410	4,305
March	5,291	3,981
April	3,514	1,301
May	3,918	700
June	5,577	1,029
July	8,675	1,632
